# Analysis of Spatiotemporal Characteristics of Pandemic SARS Spread in Mainland China

**DOI:** 10.1155/2016/7247983

**Published:** 2016-08-15

**Authors:** Chunxiang Cao, Wei Chen, Sheng Zheng, Jian Zhao, Jinfeng Wang, Wuchun Cao

**Affiliations:** ^1^State Key Laboratory of Remote Sensing Science, Beijing 100101, China; ^2^Institute of Geographic Sciences and Natural Resources Research, Chinese Academy of Sciences, Beijing 100101, China; ^3^Beijing Institute of Microbiology and Epidemiology, Beijing 100101, China

## Abstract

Severe acute respiratory syndrome (SARS) is one of the most severe emerging infectious diseases of the 21st century so far. SARS caused a pandemic that spread throughout mainland China for 7 months, infecting 5318 persons in 194 administrative regions. Using detailed mainland China epidemiological data, we study spatiotemporal aspects of this person-to-person contagious disease and simulate its spatiotemporal transmission dynamics via the Bayesian Maximum Entropy (BME) method. The BME reveals that SARS outbreaks show autocorrelation within certain spatial and temporal distances. We use BME to fit a theoretical covariance model that has a sine hole spatial component and exponential temporal component and obtain the weights of geographical and temporal autocorrelation factors. Using the covariance model, SARS dynamics were estimated and simulated under the most probable conditions. Our study suggests that SARS transmission varies in its epidemiological characteristics and SARS outbreak distributions exhibit palpable clusters on both spatial and temporal scales. In addition, the BME modelling demonstrates that SARS transmission features are affected by spatial heterogeneity, so we analyze potential causes. This may benefit epidemiological control of pandemic infectious diseases.

## 1. Introduction

In recent years, emerging infectious disease of severe acute respiratory syndrome (SARS) has become one of the most egregious public health problems in 21st-century China. SARS is caused by a new pathogen that was finally identified as a coronavirus (SARS-Cov) [1, 2]. SARS-Cov is regarded as a person-to-person infectious disease that infects suspected individuals through droplet transmission [3]. After infection, patients go through a 2-to-10-day incubation period before typical symptoms (fever, cough, and body aches) appear. During the incubation period, the patients are contagious to surrounding people [4]. To date, there is no vaccine for SARS, no reliable diagnostic test, and no specific treatment. This leads to the need for refinement of public health controls to be effective [5, 6].

Much loss of life, a high mortality rate, and substantial wealth damage were associated with SARS throughout a wide part of China in 2002 and 2003. The first case of SARS was identified and confirmed by the National Laboratory of China in Foshan, a city in Guangdong Province, South China, on November 16, 2002. With rapid development over 3 months, SARS became a disease that was difficult to control, and it spread to other cities through regular population movement [7].

According to the World Health Organization (WHO), after nearly 9 months of spread (November 16, 2002, through July 13, 2003), 29 countries had been affected by SARS. In this period, 8096 persons were infected and 774 died, which caused domestic panic in countries of East and Southeast Asia [8, 9].

The evidence shows patterns of vast geographic transmission that distinguish SARS from other epidemic infectious diseases. Traditionally, epidemiologists focus on the temporal epidemic evolution and describe it through various types of mechanical models [4, 10, 11]. Riley et al. admitted the spatial impact, but their model lacked detailed spatial analysis. However, because of the wide spread of SARS, typical temporal models have been unable to effectively explain its dynamics. As a result, the pandemic cannot be explained as a combination of endemics. Complex spatial transmission networks [12], compound period epidemics, and overwhelming government controls inspired epidemiologists to research this type of person-to-person infectious disease more profoundly with respect to its geographic features. Dye and Gay [13] suggested that doing so could lead to variations in average contact rate. Lloyd-Smith et al. simulated SARS dynamics in a complex network based on an epidemiological mechanism with full consideration of population, contact rate, control strategies, and spatial diffusion [14, 15]. However, their approach requires the assumption of numerous parameters, which in turn require the support of detailed data. The approach makes it difficult to explain overall spatial transmission without geographic epidemic analysis. Wang et al. [7] unveiled the broad existence of spatial clusters during the Beijing epidemic periods, influencing government intervention [16]. This work inspired us to investigate spatial principles in SARS transmission, which could include significant indications for pandemics within a more general perspective.

A study that combines both a temporal epidemic study and spatial transmission is required to understand SARS dynamics. Recent advances in geostatistical analysis, which have been effectively used in infectious disease mapping, health risk assessment, and epidemiological studies, could be applied in SARS research for spatiotemporal analysis [17, 18]. The Bayesian Maximum Entropy (BME) approach of modern geostatistics incorporates higher-order statistical estimation for space-time epidemic phenomena and has shown more accurate mapping results than those derived from linear kriging geostatistics [19]. We benefited from such advantages in investigating statistical features of the SARS epidemic and in simulating geographic transmission aspects on a day-by-day basis. We focused on exploring and understanding spatial heterogeneity impacts of SARS transmission and simulating and mapping the disease temporally. Under the increasing threat of pandemics in 21st century, the present study illustrates actual epidemic spread and provides useful information toward establishing government intervention to preventing pandemics.

## 2. Materials

**Figure 1:**
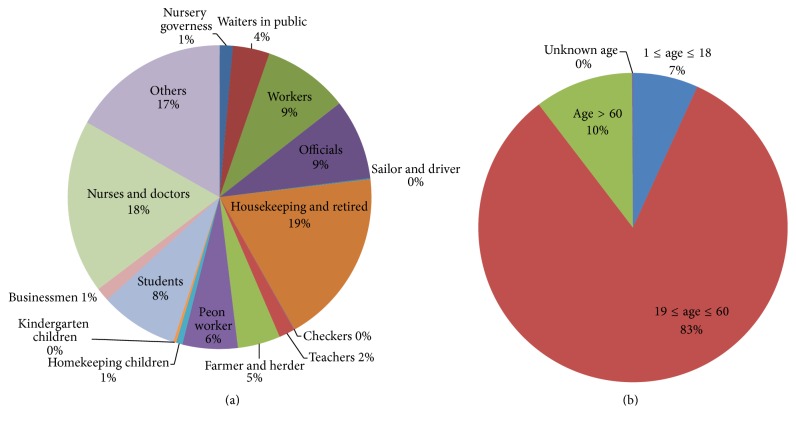
Basic patient information. (a) The occupation distribution as a percentage of all SARS infections. (b) The age percentage of SARS infections.

**Figure 2:**
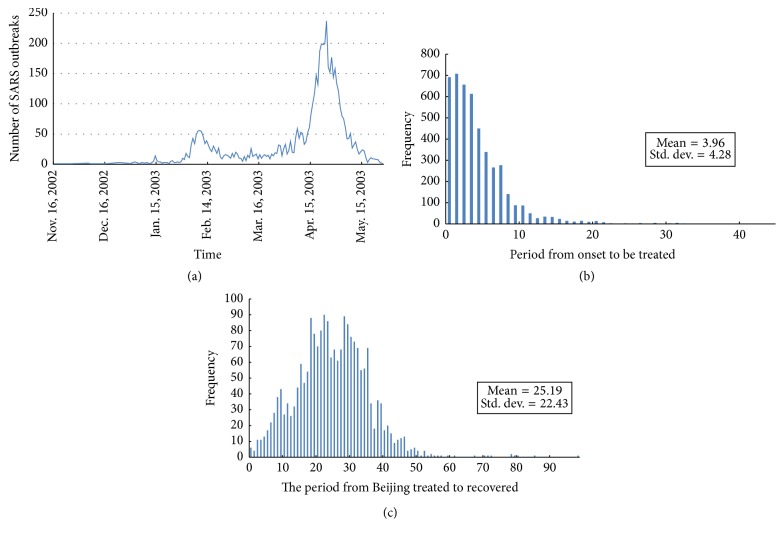
Daily number of SARS infections and temporal characteristics. (a) Number of SARS infections each day. The two outbreak peaks caused by disease spreading in Guangzhou and Beijing are clearly distinguishable in the plot. (b) Histogram of period from SARS onset to treatment (unit: day). The average period is 3.96 days, which indicates fast and rigorous health service for SARS treatment. (c) Histogram of period from treatment to recovery (unit: day). The average treatment takes 25.19 days. This period corresponds exactly to half the size of an outbreak circle.

The surveillance sanitary system of the Chinese Centre for Disease Control and Prevention (Chinese CDC) monitored all SARS infections in mainland China and confirmed them in the national microbe laboratory. The infections were recorded cautiously and totaled 5318 cases. The data were updated and include cases from November 2002 through June 2003. A confirmation system slightly different from that of WHO statistics raised the number of cases to 5327. The recorded information includes basic patient information (e.g., name, sex, age, and occupation; Figure 1), spatial information (including registered permanent residence location, company, first symptom onset location, current resident location, and hospital location), and temporal information (e.g., dates of first symptom onset, hospital treatment, recovery, or death). The daily number of SARS infections and their temporal characteristics were analyzed and are shown in Figure 2.

**Figure 3:**
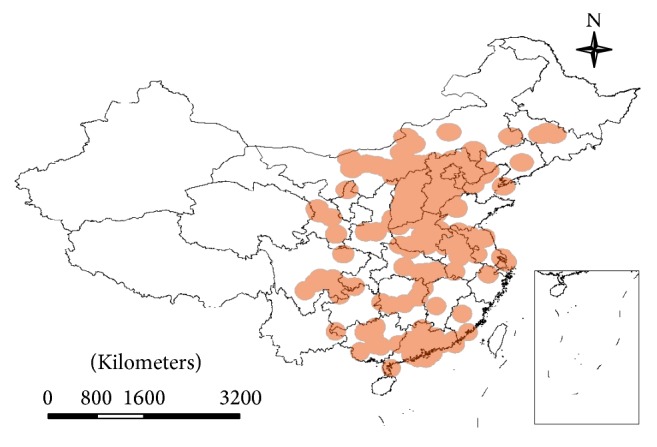
Spatial pattern of the SARS outbreaks.

In mapping all cases, the location of infection onset is an elaborate process. Even though some cases were recorded at locations using various scales, the records were eventually sorted at county level to facilitate processing. Considering that population movement is one of the effects on SARS diffusion, the following order was observed in the geocoding process: (1) registered permanent residence; (2) temporary residence (work address); (3) onset location; and (4) reporting unit location. To maintain rigorous containment, sanitary departments collected minute information for spatial indexing. Thus, the registered permanent residence was selected as the first index for the benefit of data integrity and for monitoring the impact of mass population movement. On February 1, 2003, during the period of SARS outbreaks, the Spring Festival took place. People commonly travel to join family for this traditional Chinese festival, returning to their permanent residence after 1–4 weeks. This population movement increased the probability of cross infection among family members and travellers. Consequently, with some exceptions, the temporary residence, onset, and reporting unit locations could be additional spatial information in geocoding the infections. Some cases lacked records of both permanent residence and workplace, and the only available addresses for these were syndrome onset locations. If all the above addresses were void, the hospital address was used as the only thread to locate the patient, although this was rare in our research. We focused on outbreak regions for convenience and clarity in the process. There are 194 counties in the focus area, which is described in greater detail in subsequent sections. Among them, Beijing, Guangzhou, and Taiyuan experienced the most severe SARS outbreaks. We used a 2002 county-level digital map to illustrate the epidemic (Figure 3).

In temporal mapping, we also sorted the cases chronologically. All records were sorted at daily level. The onset date of patients was set as the optimal time index. Prior to this date, there was a 2–8-day incubation period [8, 20], which is generally intractable. The period from onset to hospitalization, including the incubation period, was considered the contagion period. Because it is impossible to know the infection date accurately for a patient, incubation period data are unavailable. Therefore, onset date was the first temporal index in our research. If this date was unavailable, then the hospitalization date was used as a secondary index.

## 3. Methods

### 3.1. BME Model

Typical modes of investigation in health research focus on discovering spatial patterns or establishing a time series to study the principles that govern health phenomena. Separation of the space-time domain into spatial and temporal components permits analysis of those subdomains individually but ignores principles and correlations that may exist because of the composite spatiotemporal structure [21]. In infectious disease research, and especially the study of person-to-person contagious diseases, epidemic conditions are closely related to both spatial and temporal dimensions. Thus, proper relationships must be constructed to combine and account for the space-time continuum.

BME provides an effective stochastic method, based on a cogent theoretical and technological strategy, to analyze relationships of SARS outbreaks in the composite space-time domain. It comprises not only epidemiological knowledge bases but also spatiotemporal statistics and dynamic modelling aspects for study. It also offers a software framework for modelling and prediction of epidemic conditions across space-time [22, 23]. Details include the following.

1. BME integrates knowledge from epidemiology into Geographic Information System science by assimilating epidemic laws, empirical relations, and statistical calculations in SARS studies.
2. BME considers spatial heterogeneity in SARS outbreaks, which broadens the traditional epidemic research field from the temporal to space-time domain.
3. BME simulates SARS transmission and predicts diffusion tendency. The resultant study of these processes can reflect interesting underlying principles behind SARS spread.

For stochastic representation of the outbreaks, we considered the daily number of outbreaks as a random variable within a three-dimensional space-time random field (S/TRF) [24]. Then each of the SARS records is a single realization out of all possible values that can be observed at a specific space-time location. Two of the S/TRF dimensions correspond to geographic coordinates of the records, and the third dimension axis represents the temporal dimension. In this sense, we studied the SARS outbreaks in a composite spatiotemporal domain, in which each observed record is uniquely represented by the space-time vector *p* = (*s*, *t*). BME takes these records as individual S/TRF points, to which each is assigned a spatial location and temporal instance.

To illustrate the composite spatiotemporal approach, consider a spatial-only map at the time instance *t*_0_, represented by

(1) $$\begin{array}{c} x_{map}\left(\;0\;\right)=\left(\;x_{1},\ldots ,x_{m},x_{k1},\ldots ,x_{kn}\;\right), \end{array}$$

where [*x*_1_,…, *x*_*m*_] denotes observation points at *t*_0_ and [*x*_*k*1_,…, *x*_*kn*_] represents the estimation points at the same *t*_0_. We computed SARS distribution maps across a time interval of such *n* instances [*t*_1_,…, *t*_*n*_], and we eventually obtained a joint set of maps *p*_map_ = [*x*_map(1)_,…, *x*_map(*n*)_].

The BME method operates in three successive stages to analyze the stochastic epidemic process. In the first stage, structural characteristics of a space-time random field are incorporated in the analysis by means of all available epistemic information about the S/TRF. This information comes from theoretical or empirical sources related to the procedure and is known as the general knowledge base or G-KB. At the end of the first stage, the input allows the computation of probability density functions (PDFs) that describe the S/TRF based on the G-KB. In the case of the SARS outbreaks, we used the observations to explore general structural characteristics of the SARS S/TRF, that is, the existence of mean (or surface) trends in the space-time domain, and to explore the underlying temporal and spatial structure of the S/TRF with suitable covariance functions.

The second stage relates to the selection of case-specific information so that BME can assess characteristics and perform inference given the particular S/TRF realization facilitated by the recorded information. This information is known as the specific knowledge base or S-KB. The SARS sampling dataset that was described in the previous section consists of *m* sampling points (*p*_1_, *p*_2_,…, *p*_*m*_) and comprised the S-KB in this study.

In the final stage, BME integrates the G-KB and S-KB to compute updated prediction PDFs at selected space-time locations. The prediction or posterior PDFs provide a complete statistical description of the health attribute distribution in space-time, and they enable selection of a predictor of choice for assessment of the SARS outbreak distribution.

In traditional analysis, health attributes exhibit higher similarity for closer occurrences. Consequently, spatial distance is typically considered as the sole substantial factor that describes and demonstrates autocorrelation and the underlying disease field structure. However, the epidemic time is also important in the study of disease spread, which could extend further with rapid transportation and population movement. The previous discussion leads us to define a composite space-time distance, *dp*, as

(2) $$\begin{array}{c} \left|\;dp\;\right|=G\left(\;ds,dt\;\right), \end{array}$$

which depends on both spatial and temporal distances (*ds* and *dt*, resp.), connected through an appropriate spatiotemporal metric *G*.

Hence, the distance combined with temporal effects is more representative in epidemic analysis. In our research, *ds* = *s*_1_ − *s*_2_ and *dt* = *t*_1_ − *t*_2_ for two S/TRF points *p*_1_(*s*_1_, *t*_1_) and *p*_2_(*s*_2_, *t*_2_). Once distances are defined within the S/TRF, permissible functions can be used to describe correlations in space-time. The covariance function that yields spatial and temporal autocorrelation between two points, *p* and *p*′, is given by

(3) $$\begin{array}{c} c_{x}\left(\;p,p^{\prime }\;\right)=\bar{\left[X\left(p\right)-\bar{X\left(p\right)}\right]\left[X\left(p^{\prime }\right)-\bar{X\left(p^{\prime }\right)}\right]}. \end{array}$$

In this function, *X*(*p*) and *X*(*p*′) represent a pair of realizations (possibilities) at *p* and *p*′ in the SARS outbreak S/TRF. It is expected that the observation data sample statistics should be the same as the S/TRF. For example, the data mean model should coincide with the average statistic, and the empirical covariance model should follow the covariance statistic of the field. Let *c*_*x*_ be the arithmetic covariance. *c*_*x*_ is presumed to be a theoretical function that is the same as the traditional covariance models in kriging analysis, such as the spherical, exponential, and nugget models. Following this initial setup, the classic Bayesian model is then incorporated, as shown by

(4) $$\begin{array}{c} f\left(\;X_{k}\;|\;X_{data}\;\right)=A^{-1}\int_{D}dXf_{G}\left(\;X_{map}\;\right), \end{array}$$

where

(5) $$\begin{array}{c} A=\int_{D}dXf_{G}\left(\;X_{data}\;\right) \end{array}$$

is a normalization constant and *D* is the domain field of *X*_*k*_. In (4), note that the posterior PDF on the left side is conditioned upon the BME prior PDF, which is given in more detail by

(6) $$\begin{array}{c} f_{G}\left(\;X_{map}\;\right)=e^{\mu_{0}+\mu^{T}g\left(X_{map}\right)}=e^{\mu_{0}+\sum_{a=1}^{N}\mu_{a}g\left(X_{a}\right)}. \end{array}$$

With the support of BME, we can compute various statistical quantities, such as the mean, covariance, semivariogram, and higher-order statistics, because BME estimates the posterior PDF according to the constraints. For instance, we sought the most probable value for *X*_*k*_ and so computed the differential coefficient for *X*_*k*_ that gives the prediction PDF mode, that is,

(7) $$\begin{array}{c} \frac{\partial f\left(X_{k}\;|\;X_{data}\right)}{\partial X_{k}}=A^{-1}B\int_{D}dX\frac{\partial \sum_{a=1}^{n}\mu_{a}g\left(X_{a}\right)}{\partial X_{k}}, \end{array}$$

where *B* = *e* ^*μ*_0_^ is a constant, *g*(*X*_*a*_), *a* = 0,…, *n* are functions representing the stochastic G-KB information, and *μ*_*a*_, *a* = 0,…, *n* are the so-called Lagrange coefficients that are in spatiotemporal coordinates. The latter serve as weights for the corresponding *g*(*X*_*a*_) functions in (7). BME theory develops a system of equations for each value of *g*(*X*_*a*_), from which the *μ*_*a*_ coefficients are calculated. Subsequently, their values are inserted in (4) and (6) to evaluate the posterior PDF, whose maximum gives the prediction mode estimate.

A cross-validation procedure is followed to assess BME mapping accuracy. Specifically, we use a set of *n*_ref_ validation data that were randomly selected as references for accurate estimation. We take turns to exclude each validation datum and calculate its value at the corresponding spatiotemporal coordinates, and then we compute the mean square error (MSE), given by

(8) $$\begin{array}{c} MSE=\frac{1}{n_{ref}}\overset{n_{ref}}{\underset{i=1}{\sum }}{\left(\;X_{ref,i}-X_{i}\;\right)}^{2}. \end{array}$$

Thus, the MSE provides a measure of the uncertainty of BME estimation. In (8), *X*_ref,*i*_ is the BME estimate and *X*_*i*_ is the *i*th validation observation value.

### 3.2. Model Implementation

BME computations were performed with the specialized MATLAB-based interactive software interface known as the Spatiotemporal Epistemic Knowledge Synthesis Graphical User Interface, or SEKS-GUI [21]. SEKS-GUI is a rich-featured intuitive visual interface that provides a variety of G-KB and S-KB information types, adjusts model parameters for BME mapping, performs the analysis, and visualizes the predictions [25].

All types of data were input to the SEKS-GUI software. The original observations were sorted in a file, in which each row corresponds to a certain spatial location, date, and outbreak condition. For computing, outbreak cases recorded at county level had their spatial index converted to longitude and latitude of the corresponding county centroid. This conversion typically introduces additional uncertainty into the result, because we effectively approximate the spatial reference for each observation. But it was used in our case because these uncertainties were considered acceptable, for the following two reasons. First, all counties with SARS outbreaks were in the south and east of mainland China. These locations have high population densities and relatively small county areas. Second, the SARS infections were overwhelmingly observed in towns near their county centroids. There were 950 outbreak cases observed in 194 locations.

In addition to the previous activities, we had to define the study area. West China has only ~1% of the total national population but covers nearly half the country's area. For this reason, we refined the research area and focused on the regions 102.04°–126.56°E and 20.90°–44.12°N. The study period was November 16, 2002, to May 21, 2003—a total of 186 days.

## 4. Results

**Figure 4:**
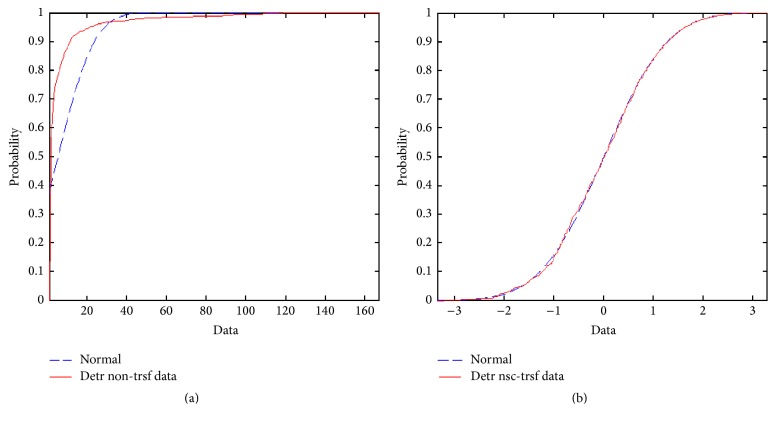
SARS data transformation. (a) Comparison of the SARS raw dataset cumulative density function (CDF) and the corresponding one of the normal distribution. The maximum observed deviation between those two CDF is about 28.88% at data value 5. (b) *N*-score transformed CDF of the SARS study data and the normal distribution. The maximum observed deviation between those two CDF has dropped down, compared to panel (a), to about 2.26% at data value −0.63853.

For statistical homogeneity, the data of outbreak numbers should have a normal distribution. In our study, the observation cumulative density function (CDF) showed as much as 36.99% deviation from the normal CDF distribution (Figure 4(a)). Thus, the original data were transformed using the normal scores method, which forces the original data distribution into the shape of the normal distribution (Figure 4(b)). Following estimation, the data are back-transformed to the original value space.

**Figure 5:**
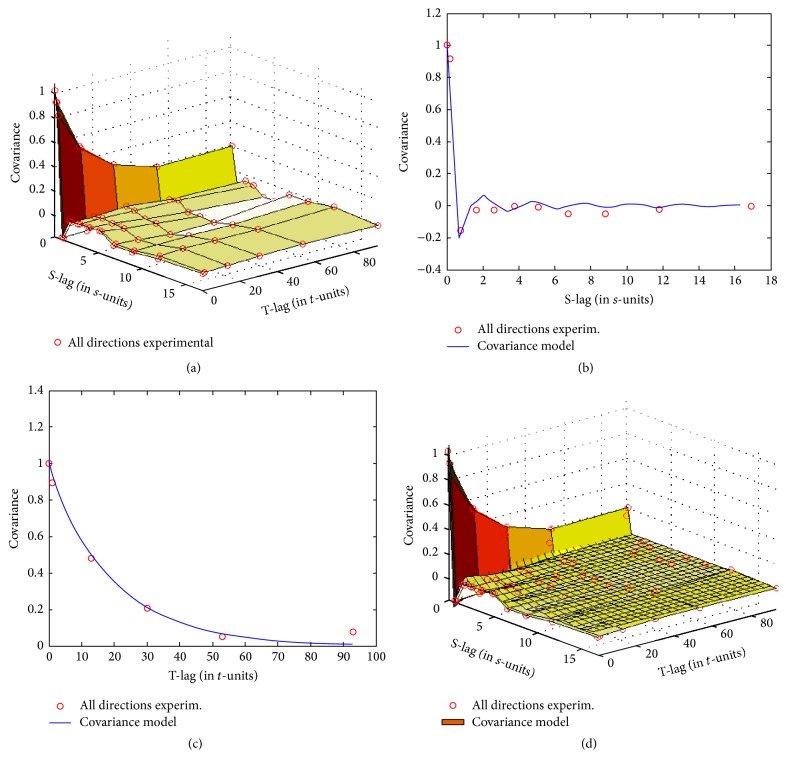
Spatiotemporal covariance used in the BME modelling. (a) Plot of the empirical spatiotemporal covariance; the covariance was estimated at the nodes shown with small red circles connected with coarse surface plates (the S-lag is in degrees and the T-lag is in days). (b) Spatial cross-section at *t* = 0 of the estimated covariance surface and the empirical covariance. (c) Temporal cross-section at *s* = 0 of the estimated covariance surface and the empirical covariance. (d) Fitted theoretical covariance plot (densely gridded surface) superimposed on the empirical covariance.

For structural correlation analysis of the spatiotemporal epidemic spread, we incorporated information from the empirical second statistical moment of the SARS S/TRF. Specifically, we computed the empirical spatiotemporal covariance and then fit a suitable permissible mathematical model to describe it for BME computation.  Figure 5 depicts both the empirical and fitted theoretical spatiotemporal covariances characterizing the SARS S/TRF.

Figure 5 shows normalized covariance values, in which darker colors correspond to higher correlation. Note that spatial correlation has some fluctuation close to zero distance. That is, covariance in space decreased rapidly and then briefly rebounded. This behavior indicates a potentially smaller-scale correlation that we could not detect with our method. Correlation in the temporal axis decreases more smoothly from its maximum value to a minimum point far from the temporal origin. Given this behavior of empirical covariance, we fit the following theoretical covariance model, which has a sine hole spatial component and exponential temporal component:

(9) $$\begin{array}{c} c_{x}\left(\;p,p\;\right)=c_{0}\exp\left(\;-\frac{3r}{as}-\frac{\sin\left(r\right)}{bt}\;\right), \end{array}$$

where *r* is distance and sin⁡(*r*) indicates a sine hole spatial component; the sill coefficient *c*_0_ is estimated at ~1.0068; the spatial lag coefficient *a* = 0.45 and the temporal lag coefficient *b* = 60.

Using the aforementioned covariance model, we obtained BME estimates representative of the pandemic-spread behavior.

The approach above allowed for a different possible explanation for the rapid decline and recovery in spatial correlation in Figure 5, as follows. A spatial lag coefficient *a* = 0.45 is in good agreement with the first geographical law; that is, the numbers of outbreak events that were <0.45 degrees in longitude (~50 km) apart had greater similarity to corresponding numbers of closer events. Inversely, there was little to no spread relationship between numbers of outbreak events >2 degrees (~220 km) apart. However, from a different perspective, two locations >220 km apart possibly showed increased correlation because of population movement between more remote locations.

The accurate fit of the exponential model to empirical temporal covariance suggests that SARS outbreak events belong in the category of traditional person-to-person epidemics. Accordingly, the specified temporal coefficient *b* of 60 days is based primarily on the duration observed in the most severely affected cities, such as Beijing, Guangzhou, and Taiyuan.

It is technically possible to limit the number of observations that are simultaneously used for BME estimation at each output grid. This is a necessary step to balance between adequate numbers of neighbors that should be used for estimation at a specific point. This is based on the assumed spatiotemporal correlation ranges on the one hand and the pragmatic need to maintain reasonable computation time for estimation, on the other. In our case study, we used up to 50 of the closest pieces of data for each estimation location. Based on the spatial lag and temporal coefficients in (9) and considerations in the previous subsection, we defined the maximum spatial and temporal ranges for neighbor search to be 15 degrees in longitude and 20 days, respectively. The parameters were set to refine calculations within reasonable estimation ranges, as follows. First, given the limited period of spread, the SARS infection presumably could not reach a location 15 degrees away within mainland China, even considering rapid population transportation by air. Then, the observed data suggest that taking the 50 pieces of data nearest an estimation point essentially considers average temporal ranges of 5 days and distances ~10 degrees. The MSE of 0.69 indicates that the BME estimation in this case was effective and the accuracy was acceptable.

Finally, we estimated the outbreaks by choosing a spatiotemporal metric parameter value of 0.3. This parameter was used to convert the space-time coordinates of any location into a common spatiotemporal distance in the continuum of SARS outbreak S/TRFs. For instance, with the above choice, the spatiotemporal distance of two outbreaks occurring at the same location 10 days apart is roughly equal to that of two simultaneous outbreaks three degrees apart. The aforementioned parameter value was based on correlation considerations and the spatiotemporal covariance in (9).

## 5. Discussion

According to our analysis, the SARS epidemic was divided into two distinct phases, namely, endemic spread and pandemic spread. The result, refined to 220 km and 60 days, shows that the SARS outbreaks were effectively captured by spatiotemporal autocorrelation. SARS is a typical person-to-person, rapidly spreading infectious disease, and it takes about 60 days to spread from an affected area to adjacent ones.

The process of outbreak spread to the adjacent areas is a stochastic one, owing to the synergistic effect of three factors. First, the SARS outbreaks were related to population density. Urban areas have higher population density than rural and residential areas, so for an outbreak in a city, it is more likely that it will spread within the urban limits than outside those limits. For example, among the total 5318 cases, 1934 were recorded in Beijing. This number is much higher than the number of outbreak cases observed in nearby cities and entire counties. Second, cities typically have convenient transportation to nearby cities and counties. Thus, commuting and population exchange between cities and nearby locations is likely to be much greater than corresponding activity between those cities and more distant areas. Consequently, suspected individuals from the nearby countryside are more likely to have travelled to a city and contacted infected people, and vice versa. This would increase the probability that the disease spreads in urban areas and their environs. The third and final factor is that control measures limit population transport, so the disease is likelier to have greater spread over nearby areas.

Following the SARS outbreaks, the Chinese government established strict control measures for quarantine of the suspect population. People travelling from affected areas were mandated to undergo several diagnostic tests during their trip. As a result, the entire transportation system worked as a rigorous diagnostic tool for potential SARS cases, and all train, airplane, and long-distance bus passengers were surveilled for the epidemic. Among the measures, individual travellers had their temperature taken. If this was above 37.5°C, the individual would be forced into a state of quarantine in isolation wards until the temperature recovered to normal levels. Such strict measures were effective in restricting outbreak spread to limited areas for each city [26, 27].

**Figure 6:**
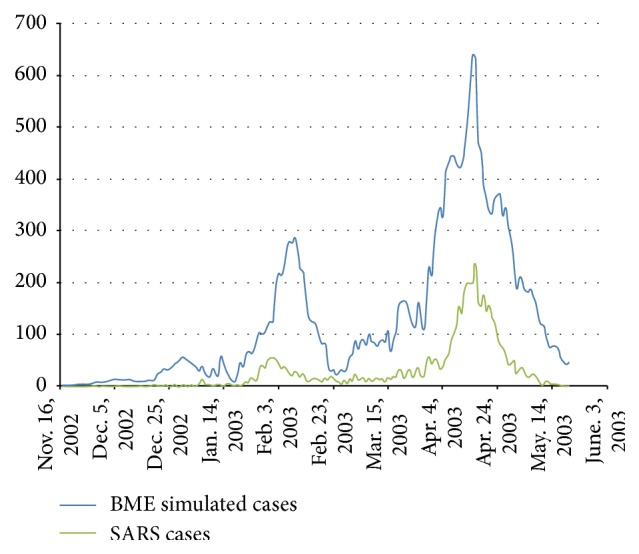
Contrast between observed SARS outbreak number and BME estimation number. The green line represents actual SARS infections on a daily basis from November 16, 2002, to May 20, 2003. The blue line represents the corresponding daily number of cases according to the BME estimates. BME reflects relatively accurately the temporal pattern in the daily number of infections, although it overestimates systematically the number values.

**Figure 7:**
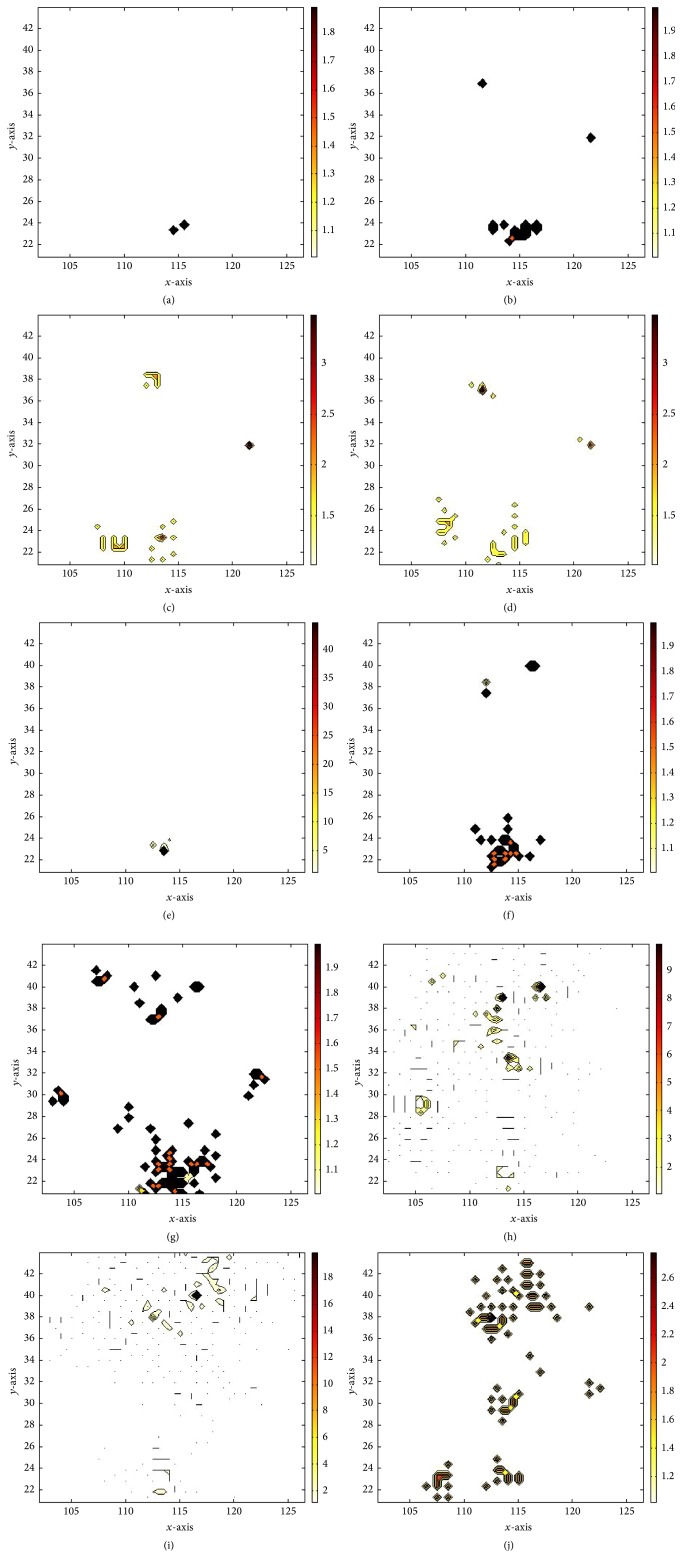
BME estimation results at selected instances. BME mode estimates are shown on (a), (b), (c), (d), (e), (f), (g), (h), (i), and (j) for those on Nov. 16, 2002, Dec. 6, 2002, Dec. 26, 2002, Jan. 15, 2003, Feb. 4, 2003, Feb. 24, 2003, Mar. 16, 2003, Apr. 5, 2003, Apr. 24, 2003, and May 20, 2003.

BME estimation led to informative results, shown in the plot of Figure 6 and the maps of Figure 7. This output reveals higher temporal correlation and relatively low spatial correlation for the SARS outbreaks and could be summarized in the following three priority rules of spreading: first, spreading occurs within the city; second, stochastic spreading occurs between the city and the towns closest to it; finally, stochastic spreading takes place across different cities through quick transportation.

The population density is an explanatory factor for the fact that SARS spread is mostly in urban areas. Approximately 19% of people working in health services were infected by their patients. These professionals were able to obtain rapid treatment, which means that it was unlikely that they would contribute to SARS spread outside their areas of residence. Housewives and retired people are groups that were extremely susceptible to infection by their families. However, these population groups typically have no desire to move to distant places. The most probable suspects for disease spread were labourers and businessmen, but these groups constituted only ~7% of total infection cases.

The randomness of SARS spread among various cities is a characteristic that has been explained via the theory of super spreaders. The starting point of this theory is that the SARS virus always evolves and mutates. Most virus strings evolve slowly with similar infectivity, but a small fraction evolve into extremely infective virus types. People affected by such virus strings are so-called super spreaders. These individuals were comparatively very contagious, and they transmitted the virus to dozens of other people. For instance, five super spreaders in Singapore were found to have infected 103 people [28, 29]. The randomness of virus mutation to high infectivity indicates considerable randomness in the way people became infected. This randomness also extended to the relatively random directions in which the epidemic spread among cities.

## 6. Conclusions

SARS generated one of the most egregious public health events in China of the beginning of 21st century, causing great loss of life and a grave threat to human survival and development. In the present study, spatial and temporal aspects of this person-to-person contagious disease were explored, and its spatial and temporal transmission dynamics were simulated through the BME method. Based on these analyses, it is concluded that SARS transmission varies in its epidemiological characteristics. Moreover, there was a high temporal correlation and relatively low spatial correlation of SARS outbreaks. In addition, the BME modelling demonstrated that SARS transmission features are affected by spatial heterogeneity. Therefore, we analyzed potential causes, including infected populations and transportation modes. Our findings can benefit epidemiological control of pandemic infectious diseases and public health protection in the future.
